# Mutational analysis of single circulating tumor cells by next generation sequencing in metastatic breast cancer

**DOI:** 10.18632/oncotarget.8431

**Published:** 2016-03-28

**Authors:** Francesca De Luca, Giada Rotunno, Francesca Salvianti, Francesca Galardi, Marta Pestrin, Stefano Gabellini, Lisa Simi, Irene Mancini, Alessandro Maria Vannucchi, Mario Pazzagli, Angelo Di Leo, Pamela Pinzani

**Affiliations:** ^1^ Sandro Pitigliani Medical Oncology Department, Hospital of Prato, Istituto Toscano Tumori, 59100, Prato, Italy; ^2^ Department of Experimental and Clinical Medicine, University of Florence, 50139, Florence, Italy; ^3^ Department of Clinical, Experimental and Biomedical Sciences, University of Florence, 50139, Florence, Italy

**Keywords:** breast cancer, circulating tumor cells, next generation sequencing, single cell sequencing, somatic mutations

## Abstract

Circulating Tumor Cells (CTCs) represent a “liquid biopsy” of the tumor potentially allowing real-time monitoring of cancer biology and therapies in individual patients.

The purpose of the study was to explore the applicability of a protocol for the molecular characterization of single CTCs by Next Generation Sequencing (NGS) in order to investigate cell heterogeneity and provide a tool for a personalized medicine approach.

CTCs were enriched and enumerated by CellSearch in blood from four metastatic breast cancer patients and singularly isolated by DEPArray. Upon whole genome amplification 3–5 single CTCs per patient were analyzed by NGS for 50 cancer-related genes.

We found 51 sequence variants in 25 genes. We observed inter- and intra-patient heterogeneity in the mutational status of CTCs.

The highest number of somatic deleterious mutations was found in the gene *TP53*, whose mutation is associated with adverse prognosis in breast cancer.

The discordance between the mutational status of the primary tumor and CTCs observed in 3 patients suggests that, in advanced stages of cancer, CTC characteristics are more closely linked to the dynamic modifications of the disease status.

In one patient the mutational profiles of CTCs before and during treatment shared only few sequence variants.

This study supports the applicability of a non-invasive approach based on the liquid biopsy in metastatic breast cancer patients which, in perspective, should allow investigating the clonal evolution of the tumor for the development of new therapeutic strategies in precision medicine.

## INTRODUCTION

Recent researches on cancer tried to explain all the characteristics of a tumor in a single individual focusing on the clinical relevance of tumor heterogeneity [1]. Notwithstanding the huge progress in elucidating cancer biology and genetics of the last decade, it is still difficult to translate into clinical practice the acquired knowledge on the emerging cellular complexity of the tumors and their dynamic features [2]. An open question in cancer biology is related to the use of appropriate tools to describe intratumoral heterogeneity and recent technologic advances in modern pathology have made it possible to analyze nucleic acids and proteins from different areas of a single tumor as well as from different cell types within the tumor, reaching the single cell resolution.

New advances in single–cell technology might help to achieve a deeper biological insight on cancer development and progression by analyzing the roles of individual cells in health and disease. Single-cell analysis is suitable to disclose information from rare cell types within a tissue or a biological fluid in order to avoid the averaging of bulk analysis and to capture the heterogeneity of cells.

Circulating tumor cells (CTCs) shed by the primary tumor as well as metastases during tumor formation and progression are now considered a real-time “liquid biopsy” reflecting the disease complexity [3]. So far the studies on CTCs have been focused on their prognostic significance, their utility in real-time monitoring of therapies, the identification of therapeutic targets and resistance mechanisms, and understanding the metastatic process [3]. Recently it has been assessed that the molecular characterization of CTCs is pivotal to increasing the diagnostic specificity of CTC assays and investigating therapeutic targets and their downstream pathways on CTCs [4].

Although we already know that CTCs are genetically heterogeneous [4–9], single-cell analysis of CTCs is the definitive and most reliable method to evidence this feature getting read of the interference from nucleated blood cells, avoiding loss of sensitivity due to the averaging that derives from the analysis of pooled samples, and understanding if the detected variants coexist in a single cell or derive from multiple cell clones [10]. Recent advances in Next Generation Sequencing (NGS) and Whole Genome Amplification (WGA) methods allow single cell analysis [11]. Single cells technologies are now playing an increasing role in the analysis of CTCs and will help the development of new therapeutic concepts in personalized medicine [12].

The presence of CTCs in early stage and metastatic breast cancer is associated with poor survival. While some authors already provided evidences for the prognostic relevance of CTCs in early breast cancer [13], data on CTCs in different subtypes of non-metastatic breast cancer are still inconsistent [14]. On the contrary, in advanced breast cancer the prognostic value of CTCs has been clearly demonstrated. In particular, CTC detection in baseline conditions has been shown as an independent predictor of progression-free survival and overall survival [15–17]. Moreover, a substantial decrease in the CTC count is an early marker of individual response to treatment and thus CTC screening provides an easy-to-perform alternative method to monitor the success of a given therapy [18].

A high number of ongoing clinical trials involve CTCs in order to evaluate the phenotype of persisting tumor cells, the benefits of secondary treatments and the survival upon additional adjuvant treatments in high risk patients [19] as well as the role of CTCs as markers of early prediction of treatment efficacy [20].

More studies are needed to clarify the clinical utility of CTC burden determination in the management of the oncologic patient [21]. To move forward in the evaluation of CTCs as surrogate biomarkers for tumor progression a further characterization of CTC biology is required, including refining and improving cell isolation methods. In fact, some contradictory results on CTCs achieved by different research groups can be attributed to the use of CTC detection methods based on several principles, varying from indirect PCR-based methods to cytomorphological identification of the tumor cells circulating in blood. Until recently, the CellSearch^®^ system has been considered as the reference method for CTC counting, but it still lacks a solution to allow a reliable downstream molecular characterization of the cells. Schneck et al. [22] proposed to recover the entire content of the CellSearch^®^ cartrigdes (including residual white blood cells) for the molecular analysis of *PIK3CA* mutations by a snapshot technique, while more recently a novel method for downstream characterization of breast cancer circulating tumor cells by a triple-immunostaining following CellSearch^®^ isolation has been published [23].

To overcome the averaging approach determined by the use of bulk CTC analysis, single cell isolation can be achieved only by sophisticated instrumentation which requires expert operators and time consuming protocols which only seldom can guarantee the single cell level. We chose a dieletrophoretic method (DEPArray™ system, Silicon Biosystems, Italy) to obtain single CTCs or pools of pure cells avoiding the inferences from leucocytes which are still present in a much higher proportion than CTCs. Recently we demonstrated the possibility of sequencing single CTCs form metastatic breast cancer patients by Sanger method [8]. The same workflow involving CellSearch^®^ enrichment followed by single cell sorting using DEPArray™ was assessed by Polzer and co-workers [24] who performed aCGH and Sanger sequencing on single CTCs from breast cancer patients.

Another approach combines the CellSearch^®^ with a flow-sorting protocol allowing genomic profiling at the single cell level [25] and demonstrating the suitability of some FACS (Fluorescence-Activated Cell Sorting) instruments for single-cell sorting.

With the present study we aim at exploring the applicability of a protocol for the molecular characterization of single CTCs by Next Generation Sequencing in order to investigate cell heterogeneity and to identify a new tool for a personalized medicine approach to patients.

In fact, in order to transfer data on CTC characterization to the clinic, the practicability and reliability of a standardized procedure represent the first issue to be verified.

## RESULTS

### Patient and tumor characteristics

Three patients had HER2 negative ER positive primary tumors and one patient had a triple negative primary breast cancer. Two patients had metastatic disease at diagnosis and were untreated before blood sampling, while the remaining two patients received five and one lines of systemic therapies for metastatic breast cancer (MBC) before blood sampling, respectively. Clinical and pathological characteristics are summarized in Table 1.

**Table 1:** Patients’ clinical and pathological characteristics

Patient ID	1	2	3	4
Tumor stage at diagnosis	IV	IV	III	III
Histology	IDC	IDC	IDC	IDC
ER status (%)	90	0	90	80
PgR Status (%)	70	0	0	80
HER2 status by IHC	0	0	1+	0
Ki67 (%)	15	40	20	30
Type of adjuvant therapy	NA	NA	CT → HT	CT → HT
DFI (months)	0	0	27	119
IMDS (months)	0	0	6	47
No. of prior lines for MBC	0	0	1	4
No. of metastatic sites	3	3	1	3
Presence of bone metastases (Y/N)	Y	N	Y	Y

Abbreviations: ER: estrogen receptor; PgR: progesterone receptor; IHC: immunohistochemistry; DFI: disease free interval (from date of breast surgery to first relapse); IMDS: interval between diagnosis of metastatic disease and blood sampling; Y: yes; N: no; MBC: metastatic breast cancer; IDC: infiltrating ductal carcinoma; CT: chemotherapy; HT: endocrine therapy; NA: not applicable.

### Quality control of the experimental procedure

The proposed workflow included several steps of quality control at the level of CTC enrichment by CellSearch^®^, whole genome amplification of single CTCs isolated by DEParray^™^ and single cell sequencing by NGS. The single steps of the quality assessment are described in the Materials and Methods section. Only samples that passed the quality controls were submitted to the subsequent analyses. Moreover we had previously verified the entire workflow on an artificial sample obtained by spiking a known number of cells from a breast cancer cell line into the blood from a healthy donor [26].

### Next Generation Sequencing on single CTCs

**Figure 1:**
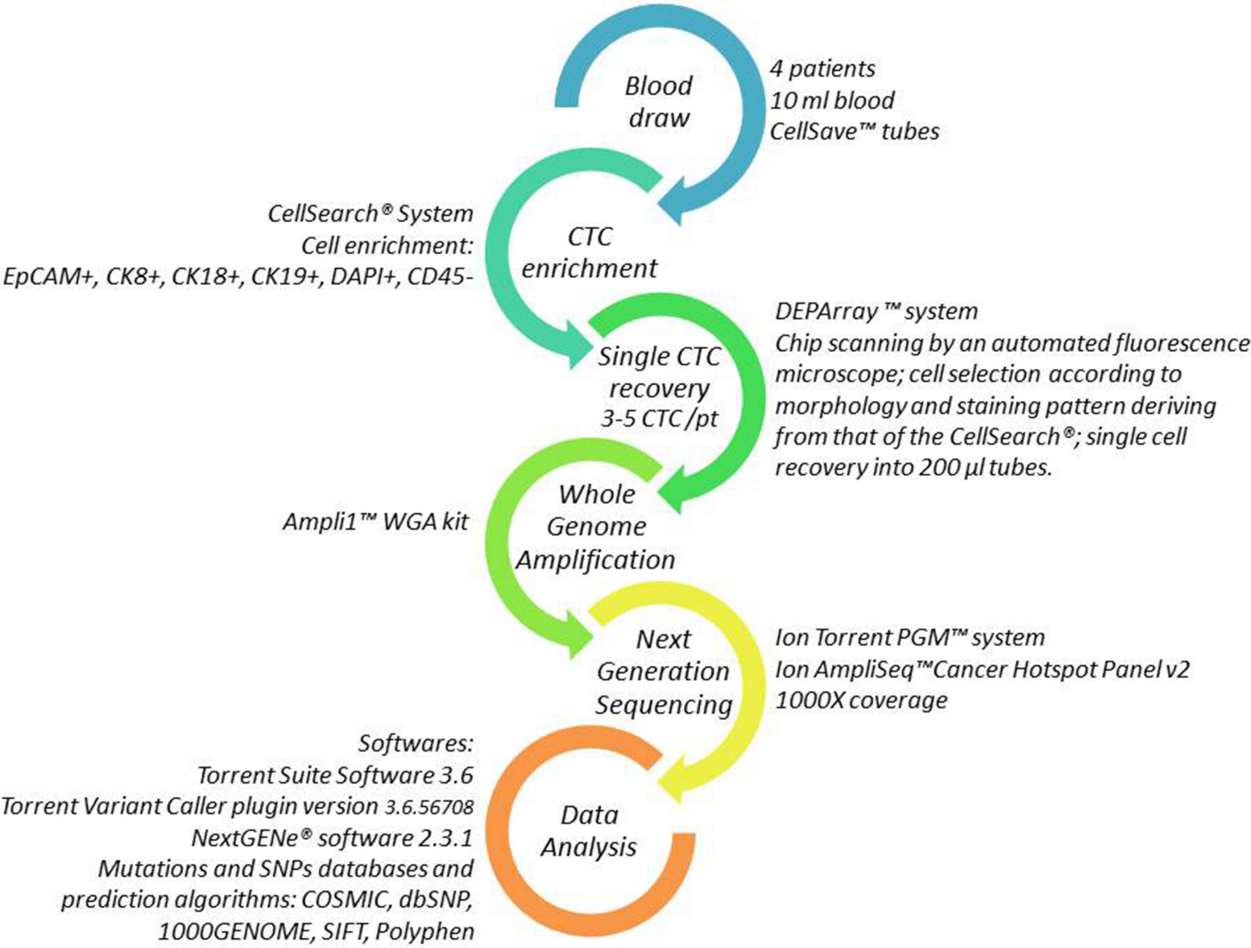
Workflow for single CTC detection and molecular analysis Each arrow represents one step of the procedure. Details on the methods are reported aside.

Results have been obtained by the experimental workflow described in details in the Materials and Methods section and illustrated in Figure 1.

**Figure 2:**
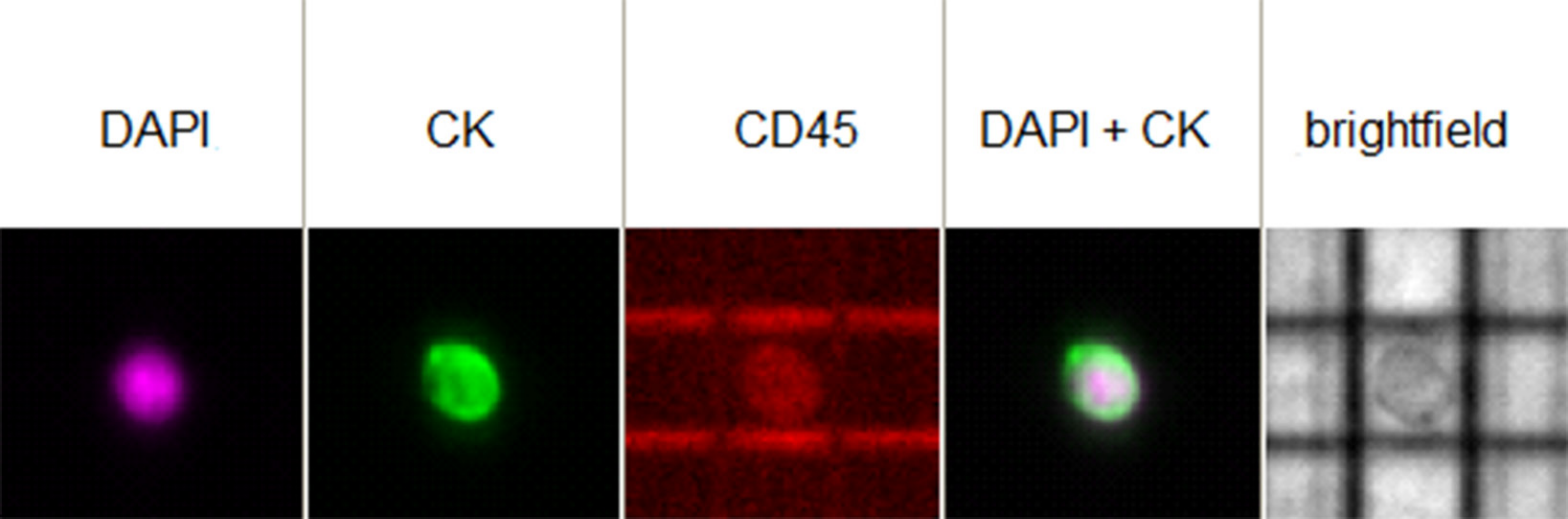
Image of a single CTC isolated from one patient as it appears in the image gallery of the DEPArray^™^ instrument The cell shows a positive fluorescent signal for DAPI (the DNA intercalating dye) and for the expression of cytokeratins (CK), while is negative for CD45.

Figure 2 reports an example of a single CTC from one of the patients of the case study as it appears in the DEPArray^™^ image gallery.

On the whole, we sequenced 14 CTCs from 4 patients with a mean depth of 1500X ranging from 1046X to 2478X depth of coverage for each amplicon per sample. According to the literature we analyzed sequence variants having at least 100X coverage [27].

**Figure 3:**
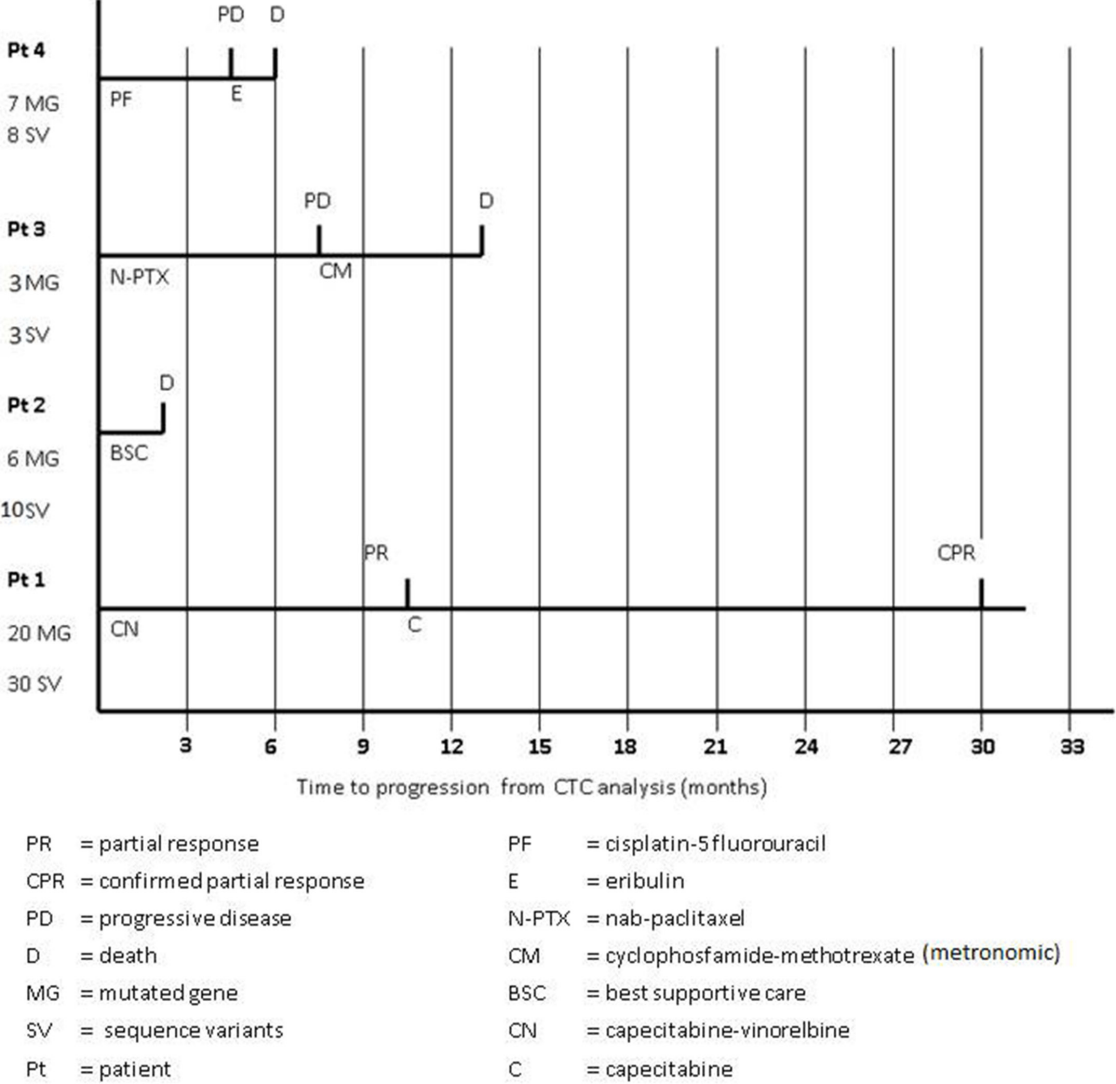
Patients’ clinical outcome from the time of blood sampling

Overall we found 51 sequence variants in 25 genes of the panel. In particular, patient 1 (the only patient for whom five CTCs were available for the analysis) showed the highest number of sequence variants (30 variants in 20 genes), while patients 2, 3 and 4 presented 10 variants in 6 genes, 3 variants in 3 genes and 8 variants in 7 genes, respectively (Table 2). Of note, patient 1, characterized by the highest number of gene mutations and sequence variants, achieved a long-lasting response to a line of chemotherapy with capecitabine-vinerolbine (Figure 3).

**Table 2:** Somatic mutations detected in single CTCs

Gene	Exon	Sequence Variant	COSMIC/HGMD	Patient 1					Patient 2			Patient 3			Patient 4		
				CTC1	CTC2	CTC3	CTC4	CTC5	CTC1	CTC2	CTC3	CTC1	CTC2	CTC3	CTC1	CTC2	CTC3
*ABL1*	7	p.S385R															
*APC*	15	p.A888S															
*ATM*	7	p.Q355X															
	7	p.A350E															
	35	p.N1801D	COSM1561127														
*BRAF*	11	p.P453H															
	15	p.D587E	COSM21609														
*CSF1R*	21	p.A960S															
*CTNNB1*	2	p.S37P	COSM5687														
*EGFR*	3	p.L119S															
*ERBB2*	20	p.V777L	COSM14062														
	20	p.P750Pfs*841															
*ERBB4*	7	p.G286V															
*FGFR2*	6	p.T268K															
*FLT3*	16	p.E672X															
*GNAQ*	5	p.Q237X															
	5	p.E234G															
*KIT*	10	p.V540V															
	10	p.M541L	COSM28026														
	10	p.K546K	COSM21983														
	11	p.E583K	CM920392														
*MLH1*	12	p.A380A															
	12	p.L393I															
*PDGFRA*	17	p.R822H	COSM447945														
	17	p.G838C															
	17	p.S851X															
	17	p.V824V	COSM22413														
*PIK3CA*	4	p.N345D	COSM1420788														
	18	p.H917N															
*PTEN*	5	p.V119A	COSM1349517														
	7	p.M264V															
	7	p.R234L	COSM540238														
*PTPN11*	3	p.G68W															
	13	p.Q506H															
*RB1*	15	p.S758P	CM050315														
*SMAD4*	11	p.P511Q															
*SMARCB1*	9	c.1119-41G>A intr	COSM 1090														
*SMO*	11	p.E208X															
	9	p.K539N															
*SRC*	3	p.E527D															
*TP53*	1	p.D7Y															
	3	p.A78S	COSM219129														
	3	p.P92H															
	4	p.P152P	COSM44061														
	4	p.H179R	COSM10889														
	5	p.A189_P190>X	COSM45186														
	5	p.Q192X	COSM10733														
	6	p.I251fs*94 intr	COSM44064														
	6	p.Y234C	COSM10725														
	7	p.R273C	COSM99933														
*VHL*	3	p.P172T															

**Figure 4:**
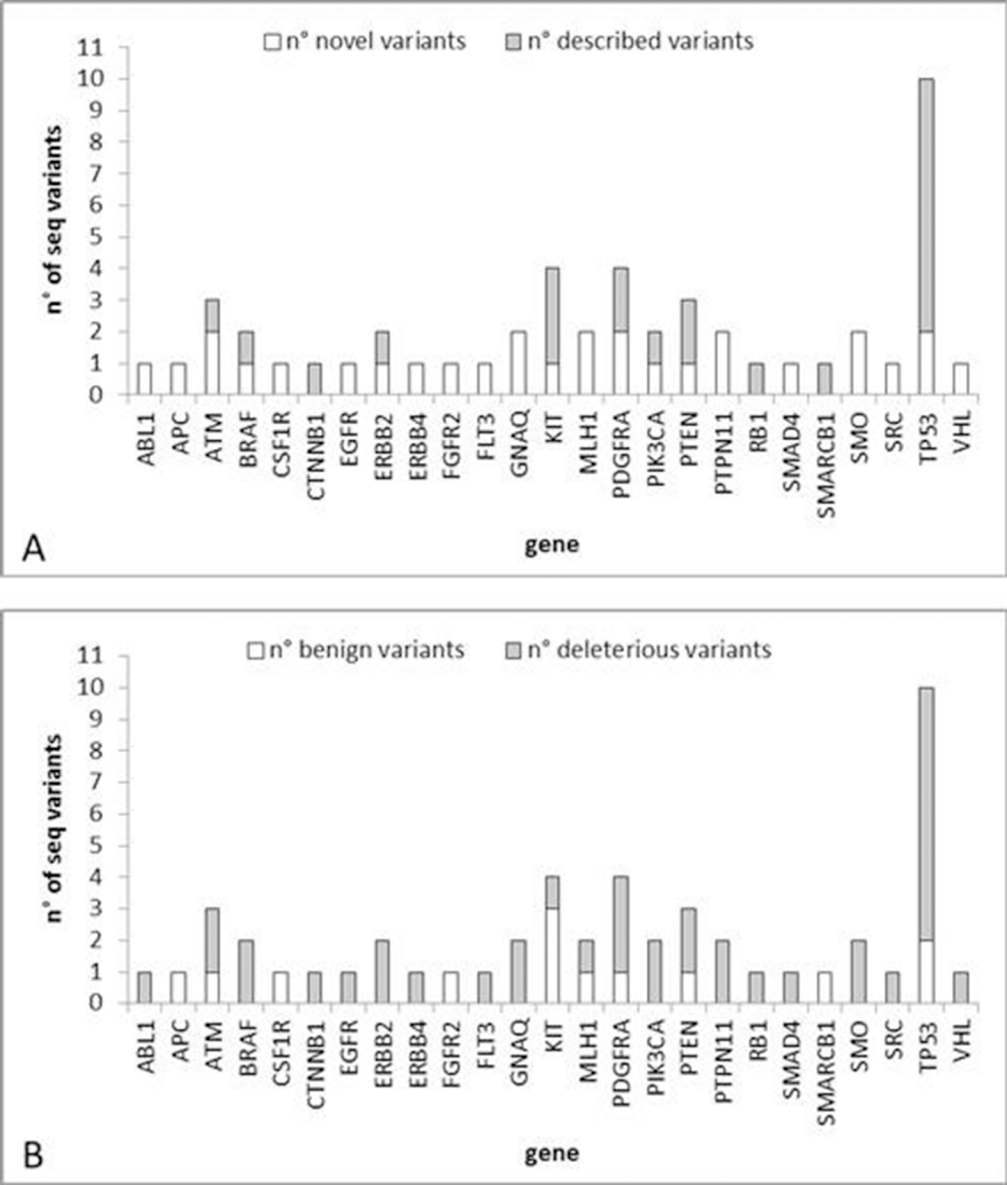
Number of sequence variants identified in the case study (**A**) Number of novel/described variants per gene. (**B**) Number of deleterious/benign variants per gene.

Among the 51 identified sequence variants, 22 were already described in the COSMIC or HGMD databases, while 29 have never been reported before (Figure 4A). Table 2 reports the sequence variants found in each CTC. Thirty-eight variants were classified as having possible deleterious consequences on the protein phenotype according to the Polyphen-2 or SIFT software, while 13 were supposed benign (Figure 4B).

The gene with the highest number of deleterious somatic mutations was *TP53* (8 mutations) followed by *PDGFRA* (3 mutations).

No sequence variant was common to different patients. The major part of somatic mutations was usually detected in only 1 single CTC from 1 patient revealing a high intra- and inter- patient heterogeneity (Table 2). Only the following sequence variants were present in two or more CTCs from the same patient:

- p.V777L in exon 20 of the *ERBB2* gene;
- p.A189_P190 > X in exon 5 of the *TP53* gene;
- p.Q192X in exon 5 of the *TP53* gene;
- p.R273C in exon 7 of the *TP53* gene;
- p.M541L in exon 10 of the *KIT* gene;
- p.V824V in exon 17 of the *PDGFRA* gene.

Among the variants listed above we selected those found in the *ERBB2* and *TP53* genes for a validation by Sanger sequencing. All the variants evidenced by NGS were confirmed by Sanger on the same samples.

### Comparison between CTCs and primary tissues

In three patients we could compare the NGS results from CTCs with those from the primary tissue (Table 3) limited to the variants found in the single CTCs.

**Table 3:** Comparison between CTCs and tissues

		Patient 2		Patient 3		Patient 4	
Gene	Exon	CTC	TISSUE	CTC	TISSUE	CTC	TISSUE
ATM	35	p.N1801D	wt	wt	wt	wt	wt
CTNNB1	2	wt	wt	wt	wt	p.S37P	wt
ERBB2	20	wt	wt	wt		p.P750Pfs*841	wt
ERBB4	7	wt	wt	wt	wt	p.G286V	wt
FGFR2	6	wt	wt	p.T268K	wt	wt	wt
GNAQ	5	wt	wt	p.Q237X	wt	p.E234G	wt
MLH1	12	p.L393I	wt	wt		wt	wt
PDGFRA	17	p.V824V	p.V824V	wt	wt	wt	wt
PIK3CA	4	wt	wt	wt	wt	p.N345D	wt
PTEN	5	wt	wt	p.V119A	p.G129R	wt	wt
PTEN	7		wt	wt	wt	p.R234L	wt
PTPN11	13	p.Q506H	wt	wt	wt	wt	wt
SMARCB1	9	c.1119-41G>A intr	wt	wt		wt	wt
TP53	1	p.D7Y	wt	wt		wt	wt
TP53	3	p.P92H	wt	wt		wt	wt
TP53	4	p.H179R	p.A161T p.R175R	wt	wt	wt	wt
TP53	5	p.A189_P190>X	p.A189_P190>X	wt		wt	wt
TP53	5	p.Q192X	p.Q192X	wt		wt	
TP53	6	wt	wt	wt	wt	p.Y234C	wt
TP53	7	wt	wt	wt		p.R273C	p.R273C

In patient 2 the benign variant p.V824V in the *PDGFRA* gene was found in both CTCs and FFPE tissue.

The only deleterious somatic mutations detected in both CTCs and tissue were the p.R273C in the *TP53* gene in patient 4 and p.A189_P190 > X plus p.Q192X in exon 5 of the *TP53* gene in patient 2. All the other variants discovered in CTCs were not present in the corresponding primary tissue.

### Comparison between CTCs isolated before and after treatment

In patient 1 a second blood sample, drawn 1 month after the start of treatment, was analysed, allowing a comparison of the mutational profile of CTCs before and during treatment (Table 4).

**Table 4:** Sequence variants detected in single CTCs from patient 1 before and while on treatment

Gene	Exon	Seq Variant	COSMIC	pt. 1 PRE-treatment					pt. 1 on treatment		
				ctc1	ctc2	ctc3	ctc4	ctc5	ctc6	ctc7	ctc8
ABL1	7	p.S385R									
APC	15	p.A888S									
ATM	7	p.Q355X									
ATM	7	p.A350E									
BRAF	11	p.P453H									
BRAF	15	p.D587E	COSM21609								
CSF1R	21	p.A960S									
EGFR	3	p.L119S									
EGFR	7	p.P281T									
ERBB2	20	p.V777L	COSM14062								
ERBB4	9	p.V348L	COSM48365								
FGFR2	6	p.K313E									
FLT3	16	p.E672X									
KIT	10	p.V540V									
KIT	10	p.M541L	COSM28026								
KIT	10	p.K546K	COSM21983								
KIT	11	p.E583K	CM920392								
MLH1	12	p.A380A									
PDGFRA	17	p.R822H	COSM447945								
PDGFRA	17	p.G838C									
PDGFRA	17	p.S851X									
PIK3CA	4	p.S326Y									
PIK3CA	18	p.H917N									
PTEN	7	p.M264V									
PTPN11	3	p.G68W									
RB1	15	p.S758P	CM050315								
SMAD4	11	p.P511Q									
SMAD4	11	p.H528R									
SMARCB1	3	p.D172G									
SMARCB1	3	p.L191L									
SMO	11	p.E208X									
SMO	9	p.K539N									
SRC	3	p.E527D									
TP53	3	p.A78S	COSM219129								
TP53	4	p.P152P	COSM44061								
TP53	6	p.I251fs*94 intr	COSM44064								
VHL	3	p.C162R	COSM18073								
VHL	3	p.P172T									

Only two sequence variants, p.V777L in *ERBB2* and p.M541L in *KIT*, were detected before and during therapy in all the analysed CTCs. All the other variants described in patient 1 at baseline were not detected while on treatment.

The following 8 somatic mutations were detected in CTCs only while on treatment:

- p.P281T in exon 7 of the *EGFR* gene;
- p.V384L in exon 9 of the *ERBB4* gene;
- p.K313E in exon 6 of the *FGFR2* gene;
- p.S326Y in exon 4 of the *PIK3CA* gene;
- p.H528R in exon 11 of the *SMAD4* gene;
- p.D172G and p.L191L in exon 3 of the *SMARCB1* gene;
- p.C162R in exon 3 of the *VHL* gene.

All the mutations were identified in only one out of three CTCs.

## DISCUSSION

Our study represents an implementation of a workflow for the molecular characterization of single CTCs by massive parallel sequencing.

Our purpose was to perform a pilot study on a limited number of samples to assess the applicability of the analysis of a panel of genes by NGS in single circulating tumor cells from breast cancer patients.

The proposed approach, combining CellSearch and DEPArray, had previously been applied to samples from cancer patients by Polzer and coworkers [24], Fernandez et al. [7] and ourselves [8] but had never been tested before for isolating single CTCs in view of a NGS analysis. Moreover, in the study by Neves et al. [25] single cells sorted by the use of a particular FACS instrument have been analyzed by aCGH which can provide evidences of chromosomal aberrations and copy number variations but is unable to perform the analysis of single point mutations as provided by the NGS approach.

The Ion AmpliSeq™ Cancer Hotspot Panel v2 is not focused on breast cancer, but it is designed as a tool to be used in any kind of cancer. Nonetheless we found several somatic mutations in *ERBB2, PIK3CA, PTEN, RB1, SMAD4* and *TP53*, genes already described as bearing driver mutations in breast cancer [28].

The highest number of somatic deleterious mutations was not surprisingly found in the tumor suppressor gene *TP53*, whose mutation is associated with adverse prognosis in breast cancer [29]. The mutation p.R273C in exon 7 of the *TP53* gene has been associated with enhanced proliferation, invasion, and drug resistance *in vitro* in breast cancer [30].

The mutation p.V777L in exon 20 of the *ERBB2* gene is already described as an activating mutation that likely drives tumorigenesis in breast cancer and it can be found in 1.6–2.0% of breast cancer patients and in about 6.0% of *ERBB2*-mutated patients [31]. When assessing sensitization to HER2-targeted therapies, cells with this mutation are highly sensitive to neratinib, but less sensitive to lapatinib, in a manner similar to wild-type *HER2*, *in vitro* [31].

Even if we studied a limited number of patients with different pathological characteristics, not allowing to draw any final conclusion on inter-patient heterogeneity, the fact that we did not identify sequence variants common to different patients may confirm that each cancer is different among individuals and underlines the importance of a personalized medicine approach to the single patient. Nonetheless, our focus was on intra-patient heterogeneity, which can explain the lack of response to targeted agents despite the presence of a given biomarker on a tumor biopsy. This is a “hot topic” for clinicians evaluating pros and cons of the metastatic site biopsy. These preliminary data seem to support the “liquid biopsy strategy” as a more appropriate tool than the “metastatic site biopsy strategy” because biomarkers assessment on individual CTCs, potentially released in the blood flow from different metastatic sites, can be more informative than biomarkers assessment on a single metastatic site biopsy.

Almost all the mutations were present in only one CTC from the same patient highlighting the importance of the analysis at the single cell level; in fact pooling the cells might provide different results not reflecting the actual heterogeneity of the CTCs. In addition rare mutations in a single CTC could be missed by a bulk analysis of the sample.

We found discordant results between the mutational status of CTCs and that of the corresponding primary tissue, probably due to the fact that in advanced stages of cancer CTCs reflect the dynamic evolution of the disease more closely than the primary tumor, even if we must take into account WGA or sequencing artifacts.

Previous studies showed that amplification and sequencing errors are a concern for single cell mutation analysis [32], but the high coverage reached for our samples makes us confident on the reliability of even minor variants found only in single samples. In fact achieving high physical coverage of the targeted sequences is crucial for calling mutations at the same regions across multiple single cells [33]. With the Ion Torrent PGM a minimum of 100–300X and 20–30X coverage is required to identify respectively insertion and deletions; as we reached a mean coverage of 1500X per amplicon, we feel confident about the reliability of the detected variants.

As far as NGS sequencing errors are concerned, a confirmation by Sanger of the identified variants can exclude false positive results, even if this laborious low throughput approach cannot be extended to all the detected variants in a study involving sequencing of a high number of genes in different samples. Accordingly, we confirmed the presence of some of the most relevant variants by Sanger sequencing on the same amplified samples. Nevertheless, we suppose that with the widespread use of NGS and deeper insights on the technical performance of the method especially at the single cell level, Sanger sequencing confirmation could be avoided.

As already showed by recently published data probing the very high accuracy and sensitivity of PGM sequencing, the error rate of the Ion Torrent is lower with respect to other NGS platforms. Anyway, due to the Ion Torrent chemistry, the homopolymer sequencing errors are known to be an issue, but the examination of our experimental results did not reveal any homopolymer-based errors or any errors caused by the position of the mutation in the amplicon. This may be due to the design of the primers used in the Cancer Hot Spot panel v2 or the location of the mutations [31–35].

However, we cannot formally exclude technical errors deriving from the WGA procedure. As already pointed out by other authors, WGA could affect subsequent sequencing results by introducing a number of technical variables such as allelic dropout, inadequate coverage, false positive and negative results [33]. For this reason we chose to adopt a WGA method which has been shown to reliably amplify the entire cellular genome homogeneously [36].

In addition we cannot exclude that the sequence variants detected in CTCs are present in minor clones of the primary tumor, thus under the detection limit of sequencing techniques, even though the use of massive parallel sequencing with a high coverage also for tissue sample analysis should have reduced the number of undetected variants. To overcome this problem we could resort to alternative methods with a higher sensitivity than sequencing techniques such as qPCR, digital PCR or COLD PCR, able to detect specific point mutations (but not suitable for detecting a higher number of variants at a time); alternatively we are convinced that analyzing a higher number of CTCs or performing different samplings of the tissue could in part solve this issue.

One of the major advantages of the liquid biopsy is the possibility of repeating the blood sample at various time points during the disease course. Our findings on patient 1 seem to indicate that CTC characterization may be applied to monitor the response to therapy; in fact CTCs collected after treatment share only few variants with those of the first blood sample, while they present some variants undetectable in baseline conditions. Interestingly the mutation p.V777L in exon 20 of the *ERBB2* gene was common to all the CTCs at baseline and in the post-therapy condition, indicating that the clone bearing this variant was resistant to the administered therapy.

On the whole our results show an extreme heterogeneity of the mutational status of single CTCs in metastatic breast cancer patients. Among the different somatic mutations we can identify druggable variants, but finding them in a single CTC raises a question about the utility of the analysis and the possibility to use the information for a therapeutic intervention. The liquid biopsy in advanced stages is representative of the complexity of the disease [3] which is difficult to treat. On the other hand monitoring the evolution of the disease from early stages by the liquid biopsy could help identifying more aggressive clones of CTCs against which target therapies could be directed.

We believe that this pilot study supports the applicability of the liquid biopsy approach in MBC patients. These results provide a rationale for further studies aiming to integrate the liquid biopsy in the context of a new generation of trials for MBC patients. Our next step will be the activation of clinical trials testing the activity of targeted therapies and correlating the response to treatment with bio-markers assessed at the CTC level. In addition, taking into account some data suggesting that gene expression profiles might be more informative in terms of pathway functional status than gene mutations [37], we are now running a pilot study aiming to evaluate both gene expression profiles and gene mutations from CTCs of MBC patients. We believe that this approach might be informative, particularly for predicting the activity of new compounds targeting the PI3K/AKT/MTOR pathway [37].

In summary, this study supports the feasibility of the liquid biopsy strategy in MBC patients and highlights the substantial intra-tumor heterogeneity occurring at the individual patient level. It is now time to incorporate the liquid biopsy approach into a new generation of trials aiming to personalize treatment of MBC patients.

## MATERIALS AND METHODS

### Patients

Ten mL of whole blood were drawn from four metastatic breast cancer (MBC) patients attending the “Sandro Pitigliani” Medical Oncology Department, Prato Hospital. Samples were drawn before starting a systemic therapy and were collected in CellSave™ tubes (Veridex LLC).

At the time of blood sampling, patients had not received any systemic treatment for at least 3 weeks. The study protocol was approved by the ethical committee of Prato Hospital and all included patients gave a written informed consent.

For patient 1, a follow-up sample of 10 ml of whole blood was drawn after one month of treatment, collected in a CellSave™ tube and processed as described below.

### Workflow for the molecular characterization of a panel of genes in single CTCs

Figure 1 reports a schematic view of the experimental workflow described below.

CTC enrichment, single CTC recovery and whole genome amplification were performed according to a protocol already described and validated in a previous paper by our research team [8]. The NGS protocol has been previously optimized by our research team on artificial samples obtained by spiking a breast cancer cell line into the blood from a healthy donor [26].

### CTC enrichment

CTC enrichment was performed by the CellSearch^®^ System. Whole blood (7.5 mL) was processed using the CellSearch^®^ Epithelial Cell kit (Veridex LLC), which selects EpCAM positive cells using ferrofluids particles coated with EpCAM antibody. The procedure involves a specific CTC quality control (CELLSEARCH^®^ CTC Control Kit consisting of 2 populations of a fixed breast cancer cell line at high and low concentration) to be performed together with patients’ samples. The quality control kit allows to verify the performance of reagents, sample processing by the CELLTRACKS^®^ AUTOPREP^®^ System, and cell analysis by the CELLTRACKS ANALYZER II^®^ System to confirm that system performance is optimal.

Cells were stained with the nuclear dye 4′,6′-diamino-2-phenylindole (DAPI), anti-cytokeratin 8, 18 and 19-phycoerythrin (PE) labelled antibodies, and CD45 antibody labelled with allophycocyanin (APC). After enrichment, isolated and stained cells were resuspended in the MagNest Device (Veridex LLC), labelled cells were analyzed in the CellTracks^®^ Analyzer II (Veridex LLC) and CTCs identified and enumerated according to the criteria specified by the manufacturer's instructions.

### Single CTC recovery

Samples enriched by CellSearch^®^ were stored protected from light at 4°C before sorting with the DEPArray™ (Silicon Biosystems). Each CTC-enriched sample was recovered from the Veridex cartridge and loaded into the DEPArray™ A300K chip (Silicon Biosystems) according to the manufacturer's instructions. The chip was set into the DEPArray™ system. Chip scanning was performed by an automated fluorescence microscope to generate an image gallery, with cells selected according to their morphology (round shape, round nucleus within the cytoplasm) and staining pattern deriving from that of the CellSearch^®^ system: DAPI positive, PE positive (CK8, CK18, CK19 positive cells), APC negative (CD45 negative cells). After CTC identification, single cells were recovered into 200 μl tubes.

### Whole Genome Amplification

Single CTCs (3–5 CTCs per patient) were submitted to Whole Genome Amplification (WGA) using the Ampli1™ WGA kit (Silicon Biosystems) according the manufacturer's instructions, in order to obtain a sample suitable for sequencing analysis.

The quality of the output product of the WGA reaction was assessed by the Ampli1™ QC kit (Silicon Biosystems) according to the manufacturer's instructions.

### Next Generation Sequencing

Sequencing analysis was performed on the Ion Torrent PGM™ system (Life Technologies, USA). Samples were amplified using the Ion AmpliSeq™ Cancer Hotspot Panel v2 (Life Technologies) designed to target 207 amplicons covering mutations from 50 oncogenes and tumor suppressor genes. DNA quantification was assessed using Qubit 2.0 Fluorometer (Life Technologies). Ten nanograms of DNA were used to prepare barcoded libraries using the Ion AmpliSeq™Library kit 2.0 and Ion Xpress™ barcode adapters (Life Technologies). The libraries were purified with Agentcourt AMPure XP (Beckman Coulter, USA) and quantified with Ion Library Quantitation Kit (Life Technologies) on StepOne Plus system (Applied Biosystems, USA).

Template preparation was performed with the Ion OneTouch™ 2 System and Ion One Touch ES. Finally sequencing was performed on PGM using Ion PGM™ Sequencing 200 kit v2 (Life Technologies) on Ion 316 chip V1. The run was set in order to achieve a 1000X coverage for each sample.

We performed a quality control (QC) of the NGS procedure according to the following steps: 1) quantification of the DNA samples through a fluorimetric assay by Qubit Fluorometer; 2) QC after the clonal amplification in the emulsion PCR, by a fluorimetric assay that determines the percentage of the Ion Sphere Particles templated samples; 3) QC by the Torrent Suite after the run, this analysis gives information about the performance of the run and the quality of the generated sequencing data.

### Data analysis

All samples were processed using the Torrent Suite Software 3.6 and variant calling was performed running the Torrent Variant Caller plugin version 3.6.56708. Moreover, samples were analyzed using the NextGENe^®^ software 2.3.1 (SoftGenetics, LLC, USA).

Each variant was investigated about its potential pathogenetic role using available gene mutations and SNPs databases and prediction algorithms (COSMIC, dbSNP, 1000GENOME, SIFT, Polyphen).

### NGS on DNA from formalin-fixed paraffin embedded (FFPE) tissues

For three patients a formalin-fixed paraffin-embedded (FFPE) primary tumor tissue block with a representative invasive part (at least 50% of cells) was available.

DNA from 10 slides of 4 μm tumor tissue sections was extracted using the FFPE Tissue kit (QIAgen, Germany). The appropriate protocol to construct the libraries according to the Ion AmpliSeq™ DNA and RNA Library Preparation manual (Revision B.0) was adopted. Sequencing was done according to the same protocol adopted for CTCs.

### Sanger sequencing

One microliter of WGA Amplified DNA was used for confirmation of selected mutations from the analyzed panel by Sanger sequencing. The sequence of the primers used for PCR reactions was the same of the Ion AmpliSeq™ Cancer Hotspot Panel v2 (Life Technologies). The reaction mixture (final volume 20 μl) contained 1X PCR Buffer, 0.8 μM dNTPs, 1 μM primers and 0.5 U HotStarTaq Plus DNA Polymerase (QIAgen, Germany). The thermal profile was: 95°C for 5 min, 40 cycles at 94°C for 30 sec, 58°C for 30 sec, 72°C for 45 sec, then 72°C for 10 min. PCR products were purified using the HiYield Gel/PCR DNA Fragments Extraction Kit (RBC Bioscience) and sequenced using the BigDye Terminator 1.1 CycleSequencing kit (Applied Biosystems). Sequence reaction was purified using ZR DNA Sequencing Clean-Up Kit (Zymo Research) and analyzed using an ABI PRISM 310 Genetic Analyzer (Applied Biosystems).

## References

[1] Shlush LI, Hershkovitz D (2015). Clonal evolution models of tumor heterogeneity. Am Soc Clin Oncol Educ Book.

[2] Greaves M, Maley CC (2012). Clonal evolution in cancer. Nature.

[3] Alix-Panabières C, Pantel K (2013). Circulating tumor cells: liquid biopsy of cancer. Clin Chem.

[4] Gasch C, Bauernhofer T, Pichler M, Langer-Freitag S, Reeh M, Seifert AM, Mauermann O, Izbicki JR, Pantel K, Riethdorf S (2013). Heterogeneity of epidermal growth factor receptor status and mutations of KRAS/PIK3CA in circulating tumor cells of patients with colorectal cancer. Clin Chem.

[5] Fabbri F, Carloni S, Zoli W, Ulivi P, Gallerani G, Fici P, Chiadini E, Passardi A, Frassineti GL, Ragazzini A, Amadori D (2013). Detection and recovery of circulating colon cancer cells using a dielectrophoresis-based device: KRAS mutation status in pure CTCs. Cancer Lett.

[6] Ni X, Zhuo M, Su Z, Duan J, Gao Y, Wang Z, Zong C, Bai H, Chapman AR, Zhao J, Xu L, An T, Ma Q (2013). Reproducible copy number variation patterns among single circulating tumor cells of lung cancer patients. Proc Natl Acad Sci U S A.

[7] Fernandez SV, Bingham C, Fittipaldi P, Austin L, Palazzo J, Palmer G, Alpaugh K, Cristofanilli M (2014). TP53 mutations detected in circulating tumor cells present in the blood of metastatic triple negative breast cancer patients. Breast Cancer Res.

[8] Pestrin M, Salvianti F, Galardi F, De Luca F, Turner N, Malorni L, Pazzagli M, Di Leo A, Pinzani P (2015). Heterogeneity of PIK3CA mutational status at the single cell level in circulating tumor cells from metastatic breast cancer patients. Mol Oncol.

[9] Sakaizawa K, Goto Y, Kiniwa Y, Uchiyama A, Harada K, Shimada S, Saida T, Ferrone S, Takata M, Uhara H, Okuyama R (2012). Mutation analysis of BRAF and KIT in circulating melanoma cells at the single cell level. Br J Cancer.

[10] Navin N, Hicks J (2011). Future medical applications of single-cell sequencing in cancer. Genome Med.

[11] Shapiro E, Biezuner T, Linnarsson S (2013). Single-cell sequencing-based technologies will revolutionize whole-organism science. Nat Rev Genet.

[12] Speicher MR (2013). Single-cell analysis: toward the clinic. Genome Med.

[13] Rack B, Schindlbeck C, Jückstock J, Andergassen U, Hepp P, Zwingers T, Friedl TW, Lorenz R, Tesch H, Fasching PA, Fehm T, Schneeweiss A, Lichtenegger W, SUCCESS Study Group (2014). Circulating tumor cells predict survival in early average-to-high risk breast cancer patients. J Natl Cancer Inst.

[14] Banys-Paluchowski M, Schneck H, Blassl C, Schultz S, Meier-Stiegen F, Niederacher D, Krawczyk N, Ruckhaeberle E, Fehm T, Neubauer H (2015). Prognostic Relevance of Circulating Tumor Cells in Molecular Subtypes of Breast Cancer. Geburtshilfe Frauenheilkd.

[15] Riethdorf S, Fritsche H, Müller V, Rau T, Schindlbeck C, Rack B, Janni W, Coith C, Beck K, Jänicke F, Jackson S, Gornet T, Cristofanilli M (2007). Detection of circulating tumor cells in peripheral blood of patients with metastatic breast cancer: a validation study of the CellSearch system. Clin Cancer Res.

[16] Cristofanilli M, Budd GT, Ellis MJ, Stopeck A, Matera J, Miller MC, Reuben JM, Doyle GV, Allard WJ, Terstappen LW, Hayes DF (2004). Circulating tumor cells, disease progression, and survival in metastatic breast cancer. N Engl J Med.

[17] Bidard FC, Peeters DJ, Fehm T, Nolé F, Gisbert-Criado R, Mavroudis D, Grisanti S, Generali D, Garcia-Saenz JA, Stebbing J, Caldas C, Gazzaniga P, Manso L (2014). Clinical validity of circulating tumour cells in patients with metastatic breast cancer: a pooled analysis of individual patient data. Lancet Oncol.

[18] Hayes DF, Cristofanilli M, Budd GT, Ellis MJ, Stopeck A, Miller MC, Matera J, Allard WJ, Doyle GV, Terstappen LW (2006). Circulating tumor cells at each follow-up time point during therapy of metastatic breast cancer patients predict progression-free and overall survival. Clin Cancer Res.

[19] Lianidou ES, Mavroudis D, Georgoulias V (2013). Clinical challenges in the molecular characterization of circulating tumour cells in breast cancer. Br J Cancer.

[20] Bidard FC, Fehm T, Ignatiadis M, Smerage JB, Alix-Panabières C, Janni W, Messina C, Paoletti C, Müller V, Hayes DF, Piccart M, Pierga JY (2013). Clinical application of circulating tumor cells in breast cancer: overview of the current interventional trials. Cancer Metastasis Rev.

[21] Gold B, Cankovic M, Furtado LV, Meier F, Gocke CD (2015). Do circulating tumor cells, exosomes, and circulating tumor nucleic acids have clinical utility?: a report of the association for molecular pathology. J Mol Diagn.

[22] Schneck H, Blassl C, Meier-Stiegen F, Neves RP, Janni W, Fehm T, Neubauer H (2013). Analysing the mutational status of PIK3CA in circulating tumor cells from metastatic breast cancer patients. Mol Oncol.

[23] Frithiof H, Welinder C, Larsson AM, Rydén L, Aaltonen K (2015). A novel method for downstream characterization of breast cancer circulating tumor cells following CellSearch isolation. J Transl Med.

[24] Polzer B, Medoro G, Pasch S, Fontana F, Zorzino L, Pestka A, Andergassen U, Meier-Stiegen F, Czyz ZT, Alberter B, Treitschke S, Schamberger T, Sergio M (2014). Molecular profiling of single circulating tumor cells with diagnostic intention. EMBO Mol Med.

[25] Neves RP, Raba K, Schmidt O, Honisch E, Meier-Stiegen F, Behrens B, Möhlendick B, Fehm T, Neubauer H, Klein CA, Polzer B, Sproll C, Fischer JC (2014). Genomic high-resolution profiling of single CKpos/CD45neg flow-sorting purified circulating tumor cells from patients with metastatic breast cancer. Clin Chem.

[26] Salvianti F, Rotunno G, Galardi F, De Luca F, Pestrin M, Vannucchi AM, Di Leo A, Pazzagli M, Pinzani P (2015). Feasibility of a workflow for the molecular characterization of single cells by next generation sequencing. Biomolecular Detection and Quantification.

[27] Tarabeux J, Zeitouni B, Moncoutier V, Tenreiro H, Abidallah K, Lair S, Legoix-Né P, Leroy Q, Rouleau E, Golmard L, Barillot E, Stern MH, Rio-Frio T (2014). Streamlined ion torrent PGM-based diagnostics: BRCA1 and BRCA2 genes as a model. Eur J Hum Genet.

[28] Stephens PJ, Tarpey PS, Davies H, Van Loo P, Greenman C, Wedge DC, Nik-Zainal S, Martin S, Varela I, Bignell GR, Yates LR, Papaemmanuil E, Beare D (2012). The landscape of cancer genes and mutational processes in breast cancer. Nature.

[29] Oliveira AM, Ross JS, Fletcher JA (2005). Tumor suppressor genes in breast cancer: the gatekeepers and the caretakers. Am J Clin Pathol.

[30] Li J, Yang L, Gaur S, Zhang K, Wu X, Yuan YC, Li H, Hu S, Weng Y, Yen Y. (2014). Mutants TP53 p.R273H and p.R273C but not p.R273G enhance cancer cell malignancy. Hum Mutat.

[31] Bose R, Kavuri SM, Searleman AC, Shen W, Shen D, Koboldt DC, Monsey J, Goel N, Aronson AB, Li S, Ma CX, Ding L, Mardis ER (2013). Activating HER2 mutations in HER2 gene amplification negative breast cancer. Cancer Discov.

[32] Heitzer E, Auer M, Gasch C, Pichler M, Ulz P, Hoffmann EM, Lax S, Waldispuehl-Geigl J, Mauermann O, Lackner C, Höfler G, Eisner F, Sill H (2013). Complex tumor genomes inferred from single circulating tumor cells by array-CGH and next-generation sequencing. Cancer Res.

[33] Navin NE (2014). Cancer genomics: one cell at a time. Genome Biol.

[34] Bolotin DA, Mamedov IZ, Britanova OV, Zvyagin IV, Shagin D, Ustyugova SV, Turchaninova MA, Lukyanov S, Lebedev YB, Chudakov DM (2012). Next generation sequencing for TCR repertoire profiling: platform-specific features and correction algorithms. Eur J Immunol.

[35] Butler KS, Young MY, Li Z, Elespuru RK, Wood SC. (2015). Performance Characteristics of the AmpliSeq Cancer Hotspot Panel v2 in combination with the Ion Torrent Next Generation Sequencing Personal Genome Machine. Regul Toxicol Pharmacol.

[36] Klein CA, Schmidt-Kittler O, Schardt JA, Pantel K, Speicher MR, Riethmüller G (1999). Comparative genomic hybridization, loss of heterozygosity, and DNA sequence analysis of single cells. Proc Natl Acad Sci U S A.

[37] Loi S, Haibe-Kains B, Majjaj S, Lallemand F, Durbecq V, Larsimont D, Gonzalez-Angulo AM, Pusztai L, Symmans WF, Bardelli A, Ellis P, Tutt AN, Gillett CE (2010). PIK3CA mutations associated with gene signature of low mTORC1 signaling and better outcomes in estrogen receptor-positive breast cancer. Proc Natl Acad Sci U S A.

